# Language Can Obscure as Well as Facilitate Apparent-Theory of Mind Performance: Part 2—The Case of Dyslexia in Adulthood

**DOI:** 10.3389/fpsyg.2021.621457

**Published:** 2021-06-24

**Authors:** Barlow C. Wright, Bernice A. L. Wright

**Affiliations:** ^1^School of Social Sciences, Nottingham Trent University, Nottingham, United Kingdom; ^2^Department of Psychology, University of Hull, Hull, United Kingdom

**Keywords:** adults, dyslexia, language, theory of mind, working memory

## Abstract

Many studies imply causal links between linguistic competencies and Theory of Mind (ToM). But despite Dyslexia being a prime example of linguistic deficits, studies on whether it is related to ToM have been relatively unforthcoming. In the first of 2 studies (*N* = 89), independently-diagnosed dyslexic adults and non-dyslexic adults were presented with false-belief vignettes via computer, answering 4 types of question (Factual, Inference, 1st-order ToM & 2nd-order ToM). Dyslexia related to lower false-belief scores. Study 2 (*N* = 93) replicated this result with a non-computer-based variant on the false-belief task. We considered the possibility that the apparent-issue with ToM is caused by processing demands more associated to domains of cognition such as language, than to ToM itself. Addressing this possibility, study 2 additionally utilised the ToM30Q questionnaire, designed largely to circumvent issues related to language and memory. Principal-Components analysis extracted 4 factors, 2 capturing perceptual/representational ToM, and the other 2 capturing affective components related to ToM. The ToM30Q was validated via its associations to a published measure of empathy, replication of the female gender advantage over males, and for one factor from the ToM30Q there was a correlation with an existing published index of ToM. However, when we considered the performance of dyslexic and non-dyslexic participants using the ToM30Q, we found absolutely no difference between them. The contrasting findings from our 2 studies here, arguably offer the first experimental evidence with adults, that there is in fact no ToM deficit in dyslexia. Additionally, this finding raises the possibility that some other groups considered in some sense atypical, failed ToM tasks, not because they actually have a ToM deficit at all, but rather because they are asked to reveal their ToM competence through cognitive domains, such as language and memory.

## Introduction

Theory of Mind (ToM) is the socio-cognitive ability to theorise about the mind as typically the cause and sometimes the target of behaviour, and the related cognitive ability to take another person's subjective perspective irrespective of whether the reasoner holds that perspective him/herself (Moran, 2013; Abdel-Hamid et al., 2019; cf. Premack and Dasser, 1991; Tompkins et al., 2013). ToM seems an important factor in social phenomena such as empathy, moral reasoning and conflict resolution (Bruneau and Saxe, 2012; Dodell-Feder et al., 2013; Gonzalez-Liencres et al., 2013). This may be partly why psychological disorders such as autism, bipolar disorder, schizophrenia, personality disorder, sensory and learning disabilities, and dementia have each been found to be associated with issues in the functioning of ToM (Gregory et al., 2002; Wolf et al., 2010; Hobson, 2014; de Vaan et al., 2018; Németh et al., 2018; Acosta et al., 2019).

Many experimental tasks for assessing ToM ultimately derive from the form of a “false-belief task” devised by Wimmer and Perner (1983). In this simple yet ingenious task, the reasoner must give a response indicating s/he understands that a person's behaviour is based on that person's subjective perception, as distinct from the reasoner's own current factual knowledge of the situation (Premack and Dasser, 1991; Lillard, 2015). Hence, such tasks tend to be termed tasks of false-belief (Wellman et al., 2001; cf. Wimmer and Perner, 1983). There are parallel profiles of ToM development across Eastern and Western cultures, however, the age at which a particular culture passes on false-belief tasks can vary by as much as 2 years (Naito, 2003). This finding was robustly confirmed in a meta-analytic comparison between 196 studies carried out in China and 155 studies carried out in the US (Liu et al., 2008). And this may impact on our ability to make precise comparisons across diverse groups when relying only on false-belief tasks.

Wellman (2018) provides an integrative account of how this “first-order” false-belief ToM ability finds its origins in more basic perceptual and social competencies, which facilitate its emergence and development during the child's first 5 years. However, “second-order” tasks demonstrate that ToM typically undergoes up to 2 more years of development before it can be said to be of similar basic maturity to ToM in adults. In second-order ToM, the reasoner contemplates the differing subjective beliefs of two protagonists in addition to his/her own current belief about a situation (Perner, 1991; Slade and Ruffman, 2005). Such higher order ToM requires appreciation and coordination of a greater number of symbolic representations and hence they highlight the importance of memory (Abell et al., 2000; Kaland et al., 2005; McKinnon and Moscovitch, 2007; Wright and Mahfoud, 2014).

The often reported finding that first- and second-order ToM are well-developed by middle childhood, could be taken to imply that adolescents and adults would perform too near ceiling for ToM tasks to be useful measures of their understandings of mind (Dodell-Feder et al., 2013). On this issue, it has been shown that if the social context of ToM reasoning is made highly relevant to situations adults might find themselves in, then second-order ToM in particular may be below ceiling even for adults (Hedden and Zhand, 2002; Keysar et al., 2003; Terwogt and Rieffe, 2003; McKinnon and Moscovitch, 2007; Im-Bolter et al., 2016). In Rutherford's (2004) task using false-belief stories, adults answered questions that involved differing beliefs of up to four protagonists (4th-order false-belief). Thus, this may have impacted on memory in addition to ToM reasoning.

Cognitive domains such as memory, executive functions and language have been confirmed to be important in ToM (Carlson and Moses, 2001; Kaland et al., 2005; Gokcen et al., 2009; Moran, 2013; Baker et al., 2014; Mary et al., 2016; Demetrious and Spanoudis, 2018). Arguably, the most important cognitive factor may be linguistic-processing (Jackson, 2001; cf. Miller, 2001; Cardillo et al., 2018; Conte et al., 2019; Bailey and Im-Bolter, 2020; Ebert, 2020; Sarmento-Henrique et al., 2020). In support of this notion, Bailey and Im-Bolter (2020) report that having epilepsy in childhood has a highly detrimental effect on ToM. Also, blind children, who tend to have an atypical language developmental trajectory in early childhood, acquire ToM some 5 years later than deaf children, who in turn acquire ToM around 2 years later than typically-developing children (Hobson, 2014; Russell et al., 1998; Peterson et al., 2000; Roch-Levecq, 2006). Given such findings regarding language and ToM in various atypical groups, we wondered about the extent to which this might generalise such that ToM performance will be impacted by any language-related developmental issue that continues into adulthood (Fahie and Symons, 2003; Kerr et al., 2003; Kaland et al., 2005; Dodell-Feder et al., 2013; Bailey and Im-Bolter, 2020).

In line with this notion, language measures taken early in childhood do tend to predict ToM performance in later childhood, much more strongly than the converse (Milligan et al., 2007). de Villiers and Pyers (2002), reported that the crucial variable for passing ToM tasks is the child's possession of more complex syntactic constructions; which have been linked to other aspects of language such as inflectional morphology, comprehension and potentially even size of vocabulary (Watson et al., 2001; Mills and Fox, 2016). Thus, notwithstanding effects of memory, language may be in some sense integral to ToM or even a prerequisite to it (cf. Astington and Jenkins, 1999; Miller, 2001; Bailey and Im-Bolter, 2020). On this language-facilitatory thesis, Bloom and German (2000) accept that if linguistic resources are in some way under-developed or compromised, this could cause failures on false-belief tasks of ToM.

However, although consistent with the idea that ToM may be predicated on language, Bloom and German's theory seems also to contemplate an alternative possible relationship: That is, language may only seem related to false-belief indexes of ToM, because we tend to test ToM using language-related protocols (e.g., syntax, vocabulary and even memory for words and spellings— Watson et al., 2001; de Villiers and Pyers, 2002; Slade and Ruffman, 2005; Mills and Fox, 2016). It may be that the more we require participants to rely on multiple symbolic representations or to have to comprehend and respond via linguistic constructions (which although perfectly grammatical may be untypical of spontaneous real world socio-cognitive interactions regarding minds), the more our participants are made to engage memory and linguistic competencies in order to tell us how they have reasoned about minds. If linguistic processes are impacted in some way, this may result in language becoming something of a barrier or obstacle to the reasoner demonstrating his/her well-developed ToM. Conversely, if we in some sense reduce the need for testing through language we might observe higher ToM performance (Bloom and German, 2000; Milligan et al., 2007; Guajardo and Cartwright, 2016).

This disadvantage (or advantage) does not have to have occurred because of atypical (or typical) linguistic development. For example, Gundel and Johnson, 2013 found that typically-developing 3 year-olds observed in their home environment demonstrate spontaneous production of sentences encapsulating ToM, even though this age group tends to fail on more formal “tests” of ToM such as via false-belief (Wellman et al., 2001; Wright and Mahfoud, 2014). Along somewhat similar lines, a deaf sub-group of children having a linguistic advantage over a second sub-group (e.g., bilingual vs. monolingual or early bilingual signers vs. late bilingual signers) tends to as a consequence demonstrate higher ToM abilities on false-belief tasks (Meristo et al., 2007).

To test between these two possibilities about ToM, one should be able to compare the ToM performance of any group experiencing significant general or specific linguistic-diversity and a second group having no such diversity. As well as allowing us to test between the linguistic-facilitatory view and the language-obscuring view of ToM, the inclusion of an appropriate language-atypical group might allow us to go even further, and test the very validity of false-belief tasks as traditionally the main tool for assessing ToM itself (Bloom and German, 2000).

On this pursuit, it is perhaps surprising to note that one salient and widely investigated developmental language disorder is conspicuous by its near-complete absence in ToM research. That disorder is dyslexia. Dyslexia is traditionally defined as a specific reading disability that is not obviously explainable by sensory impairments, general IQ or age (Jeffries and Everatt, 2004; Valdois et al., 2004; Di Filippo et al., 2008; Nandakumar and Leat, 2008; Kalyvioti and Mikropoulos, 2012). It often involves a greater deficit in spelling than in single-word reading or sentence-reading (Selikowitz, 1998; Cappelli et al., 2018). Dyslexia is also closely related to Working Memory (WM), and differences in memory can also go some way to accounting for the differing profiles of spelling (; Jeffries and Everatt, 2004; Brandenbury, 2015).

As well as reading, spelling and WM, dyslexia has recently been linked to a range of other aspects of cognition. For example, it has been linked to slower speed of processing and some deficit in production and understanding of humour and pragmatics (Pickering, 2006; Nicolson and Fawcett, 2008; Abd Ghani and Gathercole, 2013; Cappelli et al., 2018; Reis et al., 2020). Although it usually emerges fairly early in childhood, dyslexia continues to pose challenges in early adulthood and beyond, although some of these may decrease slightly with age (Reis et al., 2020). For instance, Abd Ghani and Gathercole (2013) have reported that it relates to college students' tendency towards lower academic study skills, more difficulty with time management and increased anxiety about academic performance.

Granted, some evidence seems to suggest the possibility that ToM might indeed be a factor in dyslexia (Cardillo et al., 2018). Nilsson and de Lopez (2016) found that specific language impairment (SLI), which is often taken to be similar to dyslexia, is associated with a lower ToM. More direct evidence comes from Cardillo et al. (2018). They found that children having dyslexia tended to achieve lower scores on verbally-given ToM tasks than did children not having dyslexia. However, although similar results have been obtained for young adults on pragmatic reasoning tasks (Griffiths, 2007), this finding seems yet to be replicated in ToM with an adult group having dyslexia.

### Summary of Aims and Predictions

It can be theorised that ToM might be affected by having dyslexia for two main reasons. First, it is an example of an aspect of language which might be regarded as atypical (Jeffries and Everatt, 2004; Cappelli et al., 2018). Second, if the view that language is a facilitator of ToM or even integral to it is correct, then linguistic deficits related to the accessing of-, representation of-, and maintenance of symbolic information, or the manipulation and moving between multiple linguistic forms (e.g., the representation of a past view whilst similarly representing a current view), may lead to genuine deficits in ToM. But in order to test the reality of this possible ToM deficit, it may be necessary to employ alternative tasks in addition to only using false-belief.

The present research therefore had three main aims which we addressed across two studies. Firstly, to provide an initial test of the language facilitating hypothesis of ToM against the language-obscuring hypothesis we considered above. This aim was approached by comparing a group having dyslexia to a group having no such diagnosis. We predicted that, because of the language and memory demands of the standard false-belief task, adults having dyslexia should perform less well both on first-order and second-order ToM questions than a comparison group not having dyslexia, and this finding should hold across two different variants on the false-belief task.

Secondly, we aimed to introduce an alternative way of approaching the issue of ToM measurement, that avoided as far as practicable, issues of memory, the need to set up and maintain multiple mental representations, and assessment of competencies known to be related to language. Such factors might distort measurement of the target ToM ability. The new tool introduced here assessed ToM not by measuring false-belief in terms of test scores, but rather by ascertaining self-reports about the extent to which the participants align with a variety of statements designed to be related to a number of known corollaries of ToM (e.g., own prior-belief, others' false-beliefs, interest in other minds…). If dyslexia really did involve a ToM deficit, we would have expected to find essentially the same results as in aim 1 (above).

Finally, we wanted our findings to speak to the possibility that variants on the standard false-belief task when applied to adults, may sometimes not necessarily accurately reflect the reasoner's true ToM competencies. Dyslexia being a case-in-point.

## STUDY 1

To address our first aim we designed a study which presented diagnosed dyslexic participants and non-dyslexic participants with a series of social situations told by way of short stories (vignettes) and given via written text (Tompkins et al., 2013). The vignettes were of a form used in much ToM research (e.g., McKinnon and Moscovitch, 2007) and were structured much like the stories in the Wimmer and Perner (1983) task, apart from involving situations more relevant to adults (Hedden and Zhand, 2002; Terwogt and Rieffe, 2003).

To confirm dyslexia in the dyslexic group and also to confirm no dyslexia for the control group, we additidonally took our own indices of single word reading accuracy, spelling aloud accuracy (based on the Wechsler Objective Reading Dimensions—WORD, Rust et al., 1993), and a basic measure of WM expected to be fairly independent of linguistic ability (based on a task used by Jeffries and Everatt, 2004).

### Method for Study 1

#### Participants

A total of 90 young adults studying at a UK university were assigned to one of two groups based on two main criteria. The first was a self-report of dyslexia. The second was having previously been diagnosed as having dyslexia. Diagnoses of the dyslexic group was made by professional dyslexia staff in the university student support service, with most participants having already reported a dyslexia diagnosis whilst in pre-HE education. This group was recruited on the basis of presence of dyslexia whilst having no other more pervasive language impairment on their support profile (i.e., no participants had been given a SLI/DLD diagnosis). Each participant in this group was also in receipt of student support on the basis of having dyslexia. This is an acceptable way of assigning participants to a dyslexia vs. non-dyslexia group (e.g., see meta-analysis of 178 studies by Reis et al., 2020). Indeed, only a single participant in the dyslexic group was removed because being < 1.5 SDs below the mean of the group not having dyslexia.

The resultant dyslexic group comprised 33 participants (*Mean* = 23.637 years, *SD* = 5.093), 22 of whom were female. The non-dyslexic group comprised 56 participants (*Mean* = 23.190 years, *SD* = 4.904), 35 of whom were female. The resultant total sample included in analyses was 89 participants.

#### Materials

An IBM compatible portable computer with a 2.4GHz PentiumM processor ran programs for administering the dyslexia-diagnostic tasks (i.e., reading, spelling and WM tasks) plus the critical ToM task. A second monitor was attached and responses were taken using two external devices connected to the computer. When a response was entered, the responses were immediately saved to memory.

The ToM task comprised five stories each of which outlined a particular social scenario (hereafter termed vignettes), each immediately followed by a series of eight questions in pseudo-random order (i.e., pre-randomised). The vignettes were titled Going Swimming, A Bag of Crisps, Which Shoes, Going Out and Whose Essay. The vignette “Going Out” is presented in Appendix 1, along with the corresponding questions and their categories. Full transcripts of the other vignettes are available from the first author upon reasonable request. The vignette “Going Out” involved a situation where all the characters' beliefs about what has happened are incorrect but one of these actually coincides with the current state of the world. In this vignette, three friends decide to take a break from dancing to have a drink. The main character buys two drinks with blackcurrant in them for his two friends, and a drink with coke in it for himself (he does not like blackcurrant). However, he inadvertently puts the wrong drink by his own place at the table. Then, whilst he is away for a moment, the second of the three friends swaps his own drink with the main character's drink. A factual question could ask about why the friends needed the drinks (answer = because they had done too much dancing). An inference question might ask which drink was bought for one of the main characters two friends (answer = we are only told that he buys three drinks but one is with coke because he does not like blackcurrant, so we can work the answer out inferentially). A first-order question might be about which drink the second friend will taste (answer = after switching the drinks, he believes he has the one with coke in front of him but actually his original belief was false so he has a different drink to that he thought). A second-order question might be about which drink the second friend thinks the first character believes he himself is about to drink (answer = only the participant and the first character have a true belief although for different reasons; and the second friend believes he has caused a false-belief in the main character but that is not actually correct).

Factual questions were about information directly intimated or explicitly stated in the vignette. Inference questions concerned deducible or social-contextual information that did not necessitate mentalizing. First-order questions concerned a character's belief/knowledge of a situation, which represented a currently untrue state of the world. Second-order questions concerned the participant's understanding of what the first character believes the second character believes about a situation (Duval et al., 2011).

The reason for using five vignettes was to help reduce possible fatigue effects by using vignettes that were very different from all the others. This also reduced possible practise/carryover effects, because we could limit the number of questions on each vignette to eight (two for each question type). The present data were collected and summarised automatically to give us the four categories of the ToM-related index (Factual, Inferential, 1st-order & 2nd-order), which meant we had not categorised according to total scores on each vignette. However, we computed a reliability estimate from a separate dataset (*N* = 68), which had been summarised according to vignette. The Cronbach's Alpha reliability estimate for this separate computer false-belief task was 0.937, which we considered high.

The reading task was a computer-presented variant of the reading scale of the Wechsler Objective Reading Dimensions also known as the WORD (Rust et al., 1993). This presented a total of 55 words one at a time, with these becoming progressively more challenging to pronounce correctly. The spelling task was a computer-presented variant of the Spelling Dimension of the same test. This presented the researcher with a total of 50 words plus examples of their uses in sentential contexts, which were read out for the participant to spell aloud. Both these tasks have been standardised with normally-developing individuals aged between 6 and 18 years, as well as individuals with reading and/or spelling issues (Rust et al., 1993). Thus, although the non-dyslexic participants might approach ceiling, these tests should still discriminate between dyslexic and non-dyslexic participants, and between the more and less proficient readers/spellers in the non-dyslexic group.

The primary reason for including the single-word-reading, spelling and WM tasks was to inform us about how participants' self-reported dyslexia status related to these aspects of cognition (Jeffries and Everatt, 2004; Kalyvioti and Mikropoulos, 2012). The WM task was devised to be suited for testing participants down to 5 years, so that future studies might contrast adults with young children. A monitor faced the experimenter and gave instructions for what should be presented to the participant. The experimenter read aloud a short list of previously randomly selected digits without placing any greater stress on any particular digit. For each trial, the participant waited until the list was presented and the experimenter had asked for one item from that list. The request was either for the “biggest” or the “smallest” digit (determined on a pre-randomised basis). The participant was instructed to give his/her response as quickly as possible, and only the first response was taken. This task necessitates the participant keeping the list in mind (storage aspect) and making the most basic decision about the digits in the list (mental manipulation aspect).

#### Design

A mixed factorial design was employed, both regarding reading, spelling and WM, and also regarding the four types of question from the ToM task. In each case the DV was the relevant score (e.g., reading score). For each analysis, performance was analysed as a function of group (dyslexic vs. non-dyslexic), with this dyslexia-status variable constituting the main IV.

#### Procedure

Participants were tested in a laboratory setting. The second keyboard and monitor meant that the researcher could always see the screen and start/stop the computer, and the participant had a screen which could be turned off at the appropriate times. Each participant was given the spelling task first, followed by the WM task, reading task and finally the ToM task. For the spelling task, the participant's monitor was switched off. In slight variation from the procedure presented by Rust et al. (1993) the experimenter first read out a sentence that included the word to be spelt, and then stated a single word which the participant then had to spell out aloud. All responses were audio-recorded with each participant's prior consent (McKinnon and Moscovitch, 2007). This permitted responses to be verified later on without delaying the test procedure.

For the WM task, the participant's display was again switched off. After being briefed on the task, the participant was given two examples without the computer, in which the researcher used a monotone voice to say a random series of numbers plus the prompt “biggest” or “smallest.” When the participant answered, the researcher verified it. The computer was then used to present two formal practise trials, with the participant prompted to give the answer as soon as s/he thought s/he knew what it was. Although these and other trials used digits selected earlier on a random basis, their identities and orders were now fixed. No participant had difficulty with practise trials. The 12 experimental trials were then given. These had an equal number of lists with three digits, four digits and five digits. After each trial, the researcher pressed a key to record whether the answer given had been that on the display; and then pressed a designated key to move to the next list of digits.

In the reading task, participants fixated a dot centrally on their display, and then the researcher pressed a designated key to replace the dot with the first to-be-read word. The participant read the word aloud and was then permitted to correct him/herself if s/he wished. Again correct responses were indicated by the experimenter pressing a designated key, followed by a second key to tell the computer to remove the current word and present the next word. A delay was built into the onset of each new word, which varied randomly between 1 and 2 s.

In the final section of the procedure, participants sat the ToM task. This task took the form of five blocks, one vignette per block, with each block followed by a series of eight questions. Participants read each vignette twice, to ensure they had correctly understood and remembered all the details (Schenkel et al., 2005; Russell et al., 2006).

For the first pass, the participant read the vignette silently to him/herself (Tompkins et al., 2013). This was intended to help reduce anxiety, particularly for participants who might be feeling more self-conscious about their reading.

Immediately upon finishing the currently displayed text on screen, the participant pressed the space bar. This removed the current screen. When ready, the participant pressed the space bar again to display the next screen of text. This was intended to help participants progress through the vignette at their own pace; whilst simultaneously permitting us to accurately measure the time needed to read each screen, without these times being distorted by the lengths of breaks each participant required before moving on. Please note, the reading-time data were analysed in detail and will be reported elsewhere, in order to avoid detracting from the main purpose of the present paper, and in order to be fully consistent with study 2, which would not require reading time data. However, we confirm that reading times were consistent with the group membership.

After an entire vignette had been read through once, the participant was given a break, the length of which was self determined. The vignette was then presented again in the same way as before, but this time the participant was asked to read each sentence out aloud. This allowed us to verify that the information was being read accurately; provided one condition ordering to be used elsewhere in comparisons of reading times for silent vs. reading aloud; and also helped ensure that participants had the entire vignette in mind before answering any ToM questions. Finally here, the fact that participants had already read the entire vignette through once, served to reduce any anxieties about reading aloud (Abd Ghani and Gathercole, 2013).

After a given vignette had been read twice, the test questions appeared on screen one at a time. Each question was requested via the participant pressing the spacebar. The participant read the question aloud and then answered it as soon as they felt able. The researcher had training and practise in efficiently pressing a designated key on the second keyboard as soon as the participant had pronounced the last word in the sentence, and then pressed a different key as soon as the participant began their answer. This allowed her to start and stop the computer's millisecond timer, respectively. Once the timer had been stopped, the researcher pressed a different key to signal whether the participant's response had been correct, according to an answer sheet containing acceptable answers, which was in front of her but conveniently placed out of sight of the participant. For reasons explained above, only the response-accuracy data are presented here. The entire procedure took around 45 min, excluding briefing and debriefing. Participants were thanked for their assistance and any questions they had at this time were answered.

### Results and Discussion for Study 1

For the ToM task, participants answered a total of 40 questions across five different vignettes. The questions were classified into four different question-types, factual, inference, first-order and second-order. There were two questions of each type per vignette. Across all five vignettes, the maximum number of correct responses for each question-type was 10.

Before considering the ToM data in detail, we considered the make-up of the dyslexic and non-dyslexic groups, respectively. The first indices were the tests of reading, spelling and WM. As these tests had different maximum scores (55, 50, and 12, respectively) we converted each score into a percentage for more ready comparisons (please see Appendix 2 for raw scores). The mean scores as percentages are given in Table 1 according to dyslexia-status and cognitive task.

**Table 1:** Summary of tests of spelling, WM, and reading as percentages (Study 1).

	**Spelling**	**WM**	**Reading**	**Overall**
Non-Dyslexic Female	86.659 (1.386)	94.762 (1.816)	92.727 (0.848)	91.383 (0.946)
Non-Dyslexic Male	82.635 (1.790)	93.254 (2.344)	93.680 (1.095)	89.856 (1.221)
Dyslexic Female	71.628 (1.749)	84.849 (2.290)	86.281 (1.069)	80.919 (1.193)
Dyslexic Male	72.364 (2.473)	86.364 (3.239)	83.140 (1.512)	80.623 (1.687)
Non-Dyslexic	84.647 (1.132)	94.008 (1.482)	93.203 (0.692)	90.619 (0.772)
Dyslexic	71.996 (1.514)	85.606 (1.983)	84.711 (0.926)	80.771 (1.033)
Female	79.143 (1.116)	89.805 (1.461)	89.504 (0.682)	86.151 (0.761)
Male	77.499 (1.526)	89.809 (1.999)	88.410 (0.993)	85.239 (1.041)
Overall	78.321 (0.945)	89.807 (1.238)	88.957 (0.578)	85.695 (0.645)

*Values represent percentages. Values in Parentheses are standard errors*.

Table 1 shows the spelling test tended to be more demanding than reading, with WM slightly easier than reading. It also shows a tendency for the combined average score to be almost 10% higher for the non-dyslexic group compared to the dyslexic group. A three-way Analysis of Variance (ANOVA) with gender, dyslexia-status and cognitive-task as factors, confirmed that the difference between the three diagnostic indices was statistically significant [*F*_(2, 170)_ = 54.146, *p* < 0.001, Partial Eta^2^ = 0.389, Obs.Power = 1.000]. Of note, the overall difference between our two groups was also significant [*F*_(1, 85)_ = 58.308, *p* < 0.001, Partial Eta^2^ = 0.407, Obs.Power = 1.000].

There was a 12.7% difference between the dyslexic and non-dyslexic group for spelling, reducing to 8.5% for reading and a slightly lower 8.3% for WM. This profile is in line with Reis's et al. (2020) analyses which showed WM is typically less impacted than spelling and reading. However, here, the suggested two-way interaction between dyslexia-status and cognitive-task was not statistically-significant [*F*_(2, 170)_ <1].

There was no statistically-significant overall difference according to gender as a main effect nor of gender with either dyslexia-status or cognitive task (each F < 1). The three-way interaction between gender, dyslexia-status and cognitive task was also not statistically-significant [*F*_(2, 170)_ = 1.818, *p* = 0.166, Partial Eta^2^ = 0.021, Obs.Power = 0.376].

Having confirmed our self identified dyslexic participants did show a dyslexia profile across reading, spelling and WM (Duval et al., 2011), we move on to the ToM analyses with the knowledge that our two groups may be considered indeed dyslexic and non-dyslexic, respectively. Table 2 summarises the mean scores obtained by the two respective groups for each of the four different types of questions on the ToM task—factual, inference, first-order and second-order.

**Table 2:** Summary of ToM performance by group and gender (Study 1).

	**Factual**	**Inference**	**1st-Order**	**2nd-Order**	**Overall**
Non-Dyslexic Female	8.857 (0.188)	7.971 (0.225)	7.714 (0.256)	7.314 (0.254)	7.964 (0.162)
Non-Dyslexic Male	8.762 (0.243)	7.667 (0.291)	8.667 (0.331)	7.476 (0.327)	8.143 (0.209)
Dyslexic Female	8.636 (0.237)	6.682 (0.284)	6.591 (0.323)	6.227 (0.320)	7.034 (0.204)
Dyslexic Male	8.273 (0.336)	6.455 (0.402)	6.727 (0.457)	6.455 (0.452)	6.977 (0.288)
Non-Dyslexic	8.810 (0.154)	7.819 (0.184)	8.190 (0.209)	7.395 (0.207)	8.054 (0.132)
Dyslexic	8.455 (0.206)	6.568 (0.246)	6.659 (0.280)	6.341 (0.277)	7.006 (0.177)
Female	8.747 (0.151)	7.327 (0.181)	7.153 (0.206)	6.771 (0.204)	7.499 (0.130)
Male	8.517 (0.207)	7.061 (0.248)	7.697 (0.282)	6.965 (0.279)	7.560 (0.178)
Overall	8.632 (0.128)	7.194 (0.154)	7.425 (0.175)	6.868 (0.173)	7.530 (0.110)

*Maximum possible value is 10. Values in Parentheses are standard errors*.

Table 2 shows our two groups evidenced very similar performance on factual questions. So, they had each retained the information in memory well. The relatively marked difference between the groups on ToM questions (Table 2), would therefore seem not to have resulted from differential retention of vignettes in any straightforward way.

Average performance was lower for the dyslexic group. Table 2 also shows a tendency for factual questions to attract the highest scores, followed by inference questions. First-order questions showed lower scores than inferential questions, with second-order questions hardest of all. This profile was interesting given that we had ensured the inference questions were of the same form as used for the first-order ToM questions around 50% of the time, and the same form as the second-order questions the rest of the time.

We analysed these trends using a three-way ANOVA, having factors of dyslexia-status, question-type and gender. The main effect of dyslexia-status was statistically significant [*F*_(1, 85)_ = 22.664, *p* < 0.001, Partial Eta^2^ = 0.210, Obs.Power = 0.997]. The overall difference between the question-types was also statistically significant [*F*_(3, 255)_ = 34.248, *p* < 0.001, Partial Eta^2^ = 0.287, Obs.Power = 1.000].

Paired-contrasts showed that the higher performance on factual questions compared to first-order ToM was statistically significant (*p* < 0.001). However, the slender advantage of first-order ToM compared to inference was not statistically significant (*p* = 0.233). The higher performance of first-order ToM compared to second-order ToM was statistically significant (*p* = 0.001).

The dyslexic group's profile from question-type to question-type differed significantly from that of the non-dyslexic group [two-way interaction—*F*_(3, 255)_ = 3.639, *p* = 0.013, Partial Eta^2^ = 0.041, Obs.Power = 0.794]. From Table 2 we observe that the difference between groups was smallest for the factual question, which did not necessitate inferential processing or thinking in terms of minds. However, as question-type required processing of the mental states of one and then more than one protagonist's subjective viewpoint, the difference between our two groups diverged.

Neither gender as a main effect nor the two-way or three-way interactions involving gender were statistically-significant. The two-way interaction between gender and question-type had an *F* > 1 but was not significant [*F*_(3, 255)_ = 2.134, *p* = 0.096, Partial Eta^2^ = 0.024, Obs.Power = 0.540]. All remaining interactions with gender were also non-statistically-significant (each F < 1).

## STUDY 2

Let us initially take the findings of Study 1 at face value. This invites the interpretation that dyslexia is related to a deficit in ToM (Abd Ghani and Gathercole, 2013; Cappelli et al., 2018; Cardillo et al., 2018). However, now consider our thesis that false-belief tasks require the reasoner to represent the social situation of the protagonist in mind over time, in addition to representing the reasoner's current understanding of the situation. This requires the ability to set up mental tokens for things in the real world; what Lillard and Kavanaugh (2014) call a symbolic representational capacity (see also Abell et al., 2000). Both the respective situations need to be held in memory whilst the reasoner decides which of them is required to answer the various questions on the task (Kaland et al., 2005; McKinnon and Moscovitch, 2007).

We additionally need the ability to move mentally between, and to appropriately suppress, either one of these two differing subjective perceptions/representations. For first-order ToM this is one representation on behalf of the protagonist and the other representation being of the reasoner him/herself (Leslie et al., 2004; Russell et al., 2006; Sabbagh et al., 2006; Lallier et al., 2009; Lillard and Kavanaugh, 2014). Perhaps most importantly, the appreciation of the narrative of the task and the ability to explain what is happening requires a well-developed linguistic competence, such as regarding an adequate vocabulary, for syntax or for sentence-complements (Simmons and Singleton, 2000; Miller, 2001; Ransby and Swanson, 2003; Slade and Ruffman, 2005; Moran, 2013; Cardillo et al., 2018). So, cognitive domains such as memory, attention or in particular language seem integral to ToM. However, as outlined earlier, such relationships may stem more from the nature of tasks we tend to use (false-belief performance measurements), rather than being genuine differences in ToM between the two groups (Bloom and German, 2000; Milligan et al., 2007; Guajardo and Cartwright, 2016).

For our alternative measure of ToM, we turned to a questionnaire index instead of the more experimental-task-based index such as the false-belief task used in study 1. Questionnaires have occasionally been said to be relatively unsuited for assessing ToM (Realo et al., 2003). However, here, instead of the questionnaire testing ToM directly using a score (e.g., Rutherford, 2004), we asked participants about their behaviours, feelings and dispositions towards/about themselves and other people in quite everyday situations (Chinn and Crossmann, 1995; Hales, 1995; Griffiths, 2007; Abd Ghani and Gathercole, 2013; Dodell-Feder et al., 2013). In this way, we could assess participants ToM without the need for the assessment to be confounded with memory, attention and language competencies. As this new questionnaire tool comprised 30 questions, we termed it the ToM30Q.

To assist consideration of whether the ToM30Q was valid, we considered three separate partial-validations. The first of these was against an existing written tool for indexing ToM. One of the most noted is Rutherford's (2004) ToM stories task using embedded false-belief. This task centres on stories typically involving four characters who have false beliefs about what one of the other characters believes. After reading a story, the participant considers a number of statements, each using the two-alternative forced choice response format. The participant then has to select the correct belief or factual statement from the two options. Both types of questions could ask about first-order ToM or higher order ToM, with analogous questions asked about the facts of the stories.

The Rutherford task is given in written form but it assesses ToM in a way said to be similar to the more standard false-belief task used with children (Rutherford, 2004). We expected that, if the Rutherford task is assessing the same ToM construct as our ToM30Q, we should find that a ToM factor we extract from our ToM30Q would be correlated with the Rutherford task. However, if measuring different things (e.g., ToM independent of language vs. ToM affected by language, respectively), then we would have expected such a correlation to be absent.

The second partial validation of the ToM30Q was based around the relationship between ToM and empathy (Christov-Moore et al., 2014). Decety et al. (2010) define empathy as the ability to share a wide range of emotions and feelings of others but without this stemming from direct emotional stimulation. Basically, it is the ability metaphorically to put oneself in someone else's shoes. This ability to imagine how someone else feels, is not the same as the ability to entertain a false belief, but the two competencies are generally taken to be quite closely associated (Blair, 2005; Singer, 2006).

This means that we should be able to partially validate our ToM30Q against an existing measure of empathy. The questionnaire used here was the Empathy Components Questionnaire (ECQ—Batchelder et al., 2017).

The third way of partially validating the ToM30Q was to consider whether it results in differences between certain groups of participants. For example, gender differences in ToM are quite slight during middle childhood, with the advantage tending to be for girls (Charman et al., 2002; Walker, 2005; Gardner et al., 2012; Meneghetti et al., 2012). The female advantage appears more substantial in adolescence and adulthood (Ahmed and Miller, 2011; Gardner et al., 2012; Meneghetti et al., 2012; Ibanez et al., 2013; Wacker et al., 2017), although there are some exceptions (Russell et al., 2006; Dodell-Feder et al., 2013).

Empathy, which has been previously associated to ToM, also shows up gender effects in favour of females from around 6 years of age, with the gap widening with age as for ToM (Chapman et al., 2006; Lam and Yeung, 2012).

### Method for Study 2

#### Participants

Participants were 93 adults studying or working at a UK university. They were assigned to one of two groups based on whether they reported previously being diagnosed as having dyslexia, in the same way as for study 1 earlier. The group having dyslexia comprised 25 participants of mean age 22.798 years (*SD* = 2.488, 15 females). The non-dyslexic group comprised 67 participants of mean age 24.744 years (*SD* = 5.896, 50 females). None of the participants had taken part in study 1.

#### Materials

These were the ToM30Q, the Rutherford stories task, and the Empathy Components Questionnaire. The ToM30Q contained a total of 30 questions with 24 of these intended to assess a number of hypothesised aspects of ToM but without the need for a more formal experimental test. The remaining 6 questions were intended to be control questions but were worded in a way similar to that of the ToM questions. An example is Q4—“If you are talking to someone who has tattoos, does this take your attention away from what they are saying?”. ToM questions asked about the extent to which the participant routinely considers other people's beliefs, reflects on their own past beliefs, tends to be able to read what someone is thinking based on looking at their eyes or interpreting the tone of their voice, are actually interested in what people are thinking, are easily distracted away from social interactions with other people, and consider it important to share one's beliefs with other people. An example is Q16—“When someone does something do you try to imagine what they were thinking that made them do it?”. Other questions asked about a participant's interest in recognising other people's emotional states, the participant's own emotionality, whether the participant feels they are better or not as good as their peers at telling when someone is getting upset in different circumstances, and how much they are troubled by a friend who is upset. An example is Q13—“In a face to face conversation with friends, I am one of the last to tell when someone's mood is changing” (Maszk et al., 1999).

The questionnaire both included positively worded and negatively worded questions, with the latter being reverse coded before scoring. For each question, the participant selected one of five possible responses on a Likert-type scale accompanied by semantic differentiated descriptions (Always … Never—similar to Duval et al., 2011). The full questionnaire is available upon reasonable request.

Factor Analysis was carried out after removing the six control questions. The remaining 24 questions were used to establish whether the data were consistent with one or more factors which could be identified as ToM. This analysis used the Principal Components method. Pre PCA checks demonstrated this method was appropriate. Skew and kurtosis were within +/−2.0 and +/−3.0, respectively. Also, inspection of the correlation matrix did not suggest any multi-collinearity.

Kaiser-Meyer-Olkin measure of sampling was 0.668 (i.e., above 0.6, Kaiser, 1974; Kaiser and Rice, 1974). Bartlett's test of sphericity was statistically-significant (*p* < 0.001), indicating the overall profile of correlations in the matrix differed from 0. Lastly here, communalities between items were above 0.40 (Jolliffe, 2002; Field, 2013).

PCA with orthogonal varimax factor rotation initially produced eight factors, with eigenvalues exceeding a Kaiser's criterion of 1, explaining 44.205% of the total variance. Of these eight factors, the last four contained two items or fewer. We then reran the analysis, forcing the number of factors to four, as only the first four factors had contained three or more items. This forced-factor reduction resulted in all but one of the 24 items (Q20) loading adequately onto one of the four forced factors.

Note, factor analysis was run completely independently by both investigators; yet both followed the same procedure for factor reduction and arrived at precisely the same factor structure. Additional confidence in the analysis was further boosted by preliminary analysis of a completely separate dataset based on around 400 participants but not concerning dyslexia (paper in preparation). Thus, we consider the analysis robust enough to continue.

A summary of the four factors and the rotated component matrix is given in Appendix 3. Factor 1 contained eight items, factor 2 contained seven items, factor 3 contained four items and factor 4 contained four items. The two investigators and two research assistants separately reviewed each of the four factors, in order to arrive at a consensus as to the most informative label to give each one. We tried to give a label to each, that accepted at least three of the four suggested labels, with any differences in offered labels resolved by discussion. The result of this process was that the first two factors closely related to ToM, with the other two factors more tentatively related to ToM.

The consensus label for factor 1 was “Perception-based-ToM.” This label was intended to capture the tendency for this factor to involve an interest in ToM via direct perception of eyes, voice or emotion (self-perception). This accepted prior links between ToM and emotion, as discussed by Harris (e.g., Harris, 1989; see also Hynes et al., 2006).

We called Factor 2 “mental-representational-ToM,” drawing on a phrase introduced by Perner (1991). This was largely because, irrespective of whether the emotion or the past beliefs of others were under consideration, the labels offered indicated mental representation of own and other's ToM or of own present vs. own past ToM (Coricelli, 2005). Thus, this factor reduces to a person being routinely sensitive to or interested in setting up dual representations of minds; as theoretically required, for example, by false-belief tasks (cf. Wimmer and Perner, 1983).

Factor 3, the first of the less direct indexes of ToM, was called “prioritising-the-face.” This factor seemed to revolve around an interest in being physically in the vicinity of the other person, whose subjective belief or emotional state is then interpreted by looking at the face (Harris, 1989). We called factor 4 “interest-in-others.” This factor was about how affected a person considers him/herself to be by others and, or the extent to which they find it easier to read others' beliefs if the participant already had direct experience of what the other might now be going through.

Cronbach's alpha reliability analyses were applied to the four factors. Factor 1 (perception-based-ToM) had a Cronbach's alpha value of 0.763 and this value did not increase if any one of its 8 items was excluded. For factor 2 (mental-representational-ToM), Cronbach's alpha was estimated in the same way and was 0.748, based on all 7 of its items.

For factor 3 (prioritising-the-face) we observed a Cronbach's alpha estimate of 0.626, which we considered adequate. Although the reliability estimate for factor 4 (interest-in-others) was a more moderate 0.421, we included this factor in our following analyses.

The next questionnaire was the Empathy Components Questionnaire (ECQ—Batchelder et al., 2017). This had 27 items intended to assess empathy towards other people. A factor analysis was conducted using the same procedure as for the ToM30Q. This revealed a single factor, containing 19 of the 27 items. The remaining items had low factor loadings and three or fewer items. A summary of the items loading on Factor 1 plus the items that were not robust enough to form additional factors in our particular dataset, is given in Appendix 4. Cronbach's Alpha analysis for the ECQ resulted in a reliability estimate of 0.895. We considered this again sufficient for us to proceed to data analyses proper.

The third of our tools was the Rutherford stories task. For reasons of time, we used only one of the four stories reported by Rutherford (2004). This was the story about chocolates. This task contained a story which the participant read, plus a series of nine questions. Four of the questions were control questions, and the remaining five questions were about subjective beliefs that the characters in the story held about the location of the chocolates, or the beliefs of other characters about its location. Each question was binary in form. Additionally, the ToM and control questions had highly differing difficulties by design and were converted to weighted and unweighted scores (Rutherford, 2004), rendering one or both measures non-linear. We estimated reliability based on the more linear coding of 1 point per item. We used Cronbach's Alpha and estimated the significance level via the Freedman-Chi-Square method. Computed in this way, the estimate for the present sample was 0.561, which we considered moderate.

#### Design

The design included comparisons of means on the ToM30Q according to gender, and correlations between the factors of the ToM30Q and the published index of empathy (ECQ) and ToM (Rutherford stories task). These were preceded by a preliminary analysis of the Rutherford task in terms of comparison of group means, so that we could determine if scores on this task resembled either the false-belief task profile in study 1 or more closely resembled the profile of the ToM30Q in study 2.

#### Procedure

Participants were tested in a laboratory setting, as before. They first answered a number of demographic questions, most notedly their birth sex and current gender identity. In all cases the responses of these two indexes were identical. Participants were also asked about their previous grades at GCSE level, in English, Maths, Science and Information Technology (IT). The subject having answers from virtually all participants was Science and so this subject was used to assess any academic performance differences according to group.

After the demographic questionnaire, participants completed the Rutherford stories task, the ECQ and the ToM30Q. For the ToM30Q the questions were read by the researcher to keep issues of participants' reading speed or accuracy to a minimum. For the ECQ, the same procedure was used. However, for the Rutherford task, each participant was first given 2 min to read through the story, and then the control and ToM questions were asked by the researcher as per the above questionnaires. For this tool, the participants were permitted to re-read the story as they saw fit, if this was needed to help them answer a particular question. The ToM questions were a composite of first-order and higher order questions, permitting the calculation of a raw score plus a weighted score as reported in Rutherford (2004). Altogether, this procedure took around 45 min per participant, including briefing and debriefing.

### Results and Discussion for Study 2

For the ToM30Q we calculated the average scores out of a maximum of 5, across the items of the ToM factor and also for the Emotionality factor. We did similarly for the ECQ. For the Rutherford stories task we summed the correct answers out of 5 for ToM and out of 4 for the control (non-ToM) questions. We additionally calculated the weighted scores as in Rutherford (2004).

Study 1 already showed that the self-reporting and university student support service identification of dyslexia as a diagnosis is in line with reading, spelling and WM scores. Also, in educational settings dyslexia is typically considered to be a learning disability, rather than only concerning reading difficulties (Selikowitz, 1998; Ransby and Swanson, 2003; Jeffries and Everatt, 2004; Cardillo et al., 2018). This is partly because less accurate reading, slower reading speeds or slower comprehension of what is read, can impact on learning even where the ability to read is not the primary concern of the subject (e.g., in teaching/learning mathematics—Chinn et al., 2001). Therefore, for the present study, we took a further step to look at real-life performance impacts of having dyslexia.

The first analysis here therefore considered whether the dyslexic group and non-dyslexic group showed the expected difference on GCSE science (averaged multiple/combined awards). The data were translated as follows. For the GCSE grades we scored in even numbers from A^*^ (12 points) through to grade F or lower (0 points). For example, a grade C would have a score of 6. All 25 of the group having dyslexia were entered into this analysis. However, for the non-dyslexic group, 1 of the 68 participants did not give GCSE data and so this participant's data are excluded from this initial analysis.

The mean GCSE score for the group having dyslexia was 7.920 (*SD* = 1.681), and for the non-dyslexic group the mean was 8.720 (*SD* = 1.665). In relative percentage terms, the non-dyslexic group tended to have translated science scores around 10% higher relative to the group having dyslexia. This difference is in line with the overall difference found in study 1, for reading, spelling and WM. The difference here, corresponds to just under one grade point (roughly C+ vs. B+).

A One-way Between Subjects Analysis of Variance was carried out with GCSE_Science as the dependent variable. The independent variable was dyslexia-status as in study 1. The difference between the two groups on science scores was statistically-significant [*F*_(1, 90)_ = 4.166, *p* = 0.044, Partial Eta^2^ = 0.044, Obs.Power = 0.524].

The group having dyslexia tending to have lower scores in GCSE Science (slightly less than one grade lower), is in line with research that has shown that overall, having dyslexia can impact on indexes related to academic performance (Griffiths, 2007; Abd Ghani and Gathercole, 2013; Reis et al., 2020). Thus, this first finding seems in line with the self-categorisation of the two groups as having vs. not having dyslexia.

Before turning to the ToM questionnaire data, it was considered prudent to determine whether the Rutherford stories task of ToM, distinguished between our two groups. If we are correct in our assumption that ToM tasks based around false-belief can be distorted because of their reliance on cognitive structures such as memory and language (Bloom and German, 2000), then we should find the Rutherford task intimates a ToM deficit related to dyslexia that is similar to what we found in study 1. To robustly address this question, the means for each group for control and ToM scores were calculated. These means are presented in Table 3, with the unweighted scores in the top half of the table and the weighted scores in the bottom half.

**Table 3:** Summary of main effects on the 6 variables from the Rutherford task.

	**Control**	**Theory of Mind**	**Total**
**Unweighted**			
Non-Dyslexic	3.309 (0.090)	4.500 (0.099)	7.809 (0.157)
Dyslexic	2.560 (0.149)	3.800 (0.164)	6.360 (0.259)
Overall	2.934 (0.087)	4.150 (0.096)	7.084 (0.151)
**Weighted**			
Non-Dyslexic	5.882 (0.259)	11.500 (0.298)	17.382 (0.432)
Dyslexic	4.280 (0.427)	9.200 (0.492)	13.480 (0.712)
Overall	5.081 (0.249)	10.350 (0.288)	15.431 (0.416)

*Figures in parentheses are standard errors*.

Table 3 suggests that regardless of whether we used the weighted or unweighted scores or whether we took the ToM scores, the non-ToM control scores or the total score on the Rutherford task, we saw essentially the same pattern. That is to say, the Rutherford task, just like the computer-based false-belief task of study 1, suggests a consistent tendency for participants not having dyslexia to score higher.

These data were analysed using a Multivariate Analysis of Variance (MANOVA) with dyslexia status as the between-subjects factor and the six sets of scores (2 weighting calculation modes × 3 indexes from each) as the multivariate dependent variable. The multivariate main effect of group combined across all six measures shown in Table 3, was found to be statistically-significant [Wilks' Lambda *F*_(4, 88)_ = 7.871, *p* < 0.001, Partial Eta^2^ = 0.264, Obs.Power = 0.997].

The separate analyses run for each of the six dependent variables as part of the MANOVA for the Rutherford task showed that the difference between our two groups was statistically-significant in every case [each *F*_(1, 91)_ > 13.362, *p* < 0.001, Partial Eta^2^ > 0.128, Obs.Power > 0.951]. These differences were also significant for each of the weighted Rutherford scores [each *F*_(1, 91)_ > 15.970, *p* < 0.001, Partial Eta^2^ > 0.149, Obs.Power > 0.975]. Thus, whether we considered the Rutherford scores for ToM, the scores on the control questions or even both of these combined into a total score, we obtained essentially the same finding: If we were to take test scores as our preferred index of ToM, we might well-interpret these findings as confirming that dyslexia is linked to lower ToM performance. The key findings about dyslexia in study 1 then, are unlikely to have arisen because of our use of a computer task of false-belief ToM.

So, we have confirmed that the group-wise comparison of GCSE Science scores is in line with the dyslexic vs. the non-dyslexic groups' self-reported dyslexia status, and that according to the Rutherford task there would seem to be a deficit in ToM connected to having dyslexia. However, recall our main thesis is that a relationship of language measures to ToM does not necessarily have to have arisen because language is integral to ToM. Equally, such a data profile could arise if linguistic performance measures make it harder for a participant group to report its true ToM competence. Our ToM questionnaire index of ToM does not rely on participants having to memorise fairly substantial amounts of material and does not call for mastery of particular syntactic propositional structures through which ToM reasoning must pass (Miller, 2001; de Villiers and Pyers, 2002; Milligan et al., 2007). Therefore, if one wishes to fully confirm whether the computer-based false-belief task of study 1 and the Rutherford stories task, which do agree with one another, are revealing a reality of having dyslexia, it is important to assess ToM in a way not so reliant on language/memory. This is what our ToM questionnaire is intended to address.

Turning to the three planned partial validations of the ToM30Q. A set of Pearson's correlations was run in order to determine the strength of association between the four factors (from the ToM30Q) against each of the other main variables (Rutherford stories task, ECQ Empathy, Gender). Recall from the factor analysis of this tool, the factors were labelled F1 = Perception-based-ToM; F2 = Mental-representational-ToM; F3 = Prioritising-the-face; and F4 = Sensitivity-to-others. This correlational analysis included all four factors of the ToM30Q so that we could assess whether the factors of the ToM30Q were correlated with each other. This analysis was run first with all participants included and then with only the non-dyslexic group. The results were no different in terms of statistical significance. We therefore present only the analysis with all participants included. The pairwise correlations are summarised in Table 4.

**Table 4:** Correlations between ECQ, Rutherford ToM, and ToM30Q four factors.

	**Empathy**	**Ruth ToM**	**F1**	**F2**	**F3**	**F4**	**Gender**	**Ruth CTRL**
Empathy	–	0.056 (0.296)	0.573 (<0.001)	0.561 (<0.001)	0.506 (<0.001)	0.223 (0.016)	−0.390 (<0.001)	0.051 (0.313)
Ruth ToM		–	−0.143 (0.085)	−0.032 (0.380)	0.330 (0.001)	−0.024 (0.410)	−0.111 (0.144)	0.458 (<0.001)
F1			–	0.378 (<0.001)	0.152 (0.073)	0.165 (0.057)	−0.192 (0.033)	−0.020 (0.425)
F2				–	0.294 (0.002)	0.196 (0.030)	−0.343 (<0.001)	−0.035 (0.369)
F3					–	0.237 (0.011)	−0.314 (0.001)	0.203 (0.025)
F4						–	−0.095 (0.183)	−0.251 (0.008)
Gender							–	−0.154 (0.070)
Ruth CTRL								–

*ToM, Theory of Mind. F1–F4 are factors from the ToM30Q. F1, Perceptual-based-ToM; F2, Mental-representational-ToM; F3, Prioritising-the-face; F4, Sensitivity to others; Ruth, Rutherford stories task; CTRL, control questions. Values in parentheses are significance levels for each respective pairwise correlation*.

Table 4 shows that the 4 ToM factors from the ToM30Q were significantly correlated with our measure of empathy (the ECQ). Empathy has generally been taken to form part of the basis of ToM (Dodell-Feder et al., 2013), and so the present finding of a strong association was as anticipated.

There was also a statistically-significant association between gender and the first three factors of the ToM30Q. That correlation was negative, indicating that the gender that had been coded as 1 (i.e., females) tended to have higher factor 4 scores than the gender coded as 2 (i.e., males), although it approached but did not reach statistical significance. These significant associations are again in line with findings from previous studies as regards gender and ToM in children and adults (Walker, 2005).

However, things seemed somewhat less clear for our third partial validation which was against the Rutherford stories task, intended to test for the actual application of ToM reasoning via a direct ToM score. The Rutherford ToM task was not reliably correlated with factor 1 (perception-based-ToM), factor 2 (mental-representational-ToM), nor with factor 4 (sensitivity-towards-others). However, Rutherford ToM score was correlated with factor 3 (prioritising-the-face).

Rutherford ToM was not correlated with overall empathy (via the ECQ) or with gender. This latter finding suggests that, on this occasion, the Rutherford task may not have been as good a measure of ToM as we had hoped; possibly due to us relying on only one of its stories.

That said, the finding that the Rutherford stories task was correlated with Factor 3 of the ToM30Q (prioritising-the-face), suggests that in our sample, the Rutherford story we selected was more sensitive to thinking about the facial expressions likely exhibited by the four protagonists in the story than to their hypothesised mental states.

To test the robustness of the Rutherford task and the ToM30Q as predictors of our independent index of empathy (ECQ) in the context of each other, a linear regression was carried out. This used the step-wise method in order to establish the variables most critical to predicting empathy score. We selected the backward stepping method to allow us to see how the variables are removed from the initial model (the simultaneous entry model) to settle on those variables contained in the most stable model.

This analysis produced five models, with the Rutherford ToM index the first to be removed (step 2). The final model had a multiple correlation coefficient of 0.763 [*F*_(3, 89)_ = 41.299, *p* < 0.001]; and accounted for 58.2% of the variability in the empathy index (*R*^2^ = 0.582). Table 5 shows that our final model contained the first three factors from the ToM30Q. This is further confirmation that the ToM30Q indexes phenomena that are related to empathy.

**Table 5:** Summary of final model (5) of stepwise regression onto ECQ.

**Variable name**	**Unstandardized beta**	**Standardised beta**	**Partials**	***t***	***p-*Value**
F1	0.391	0.404	0.501	5.457	<0.001
F2	0.279	0.304	0.388	3.969	<0.001
F3	0.328	0.355	0.464	4.941	<0.001

*F1, Perceptual-based-ToM; F2, Mental-representational-ToM; F3, Prioritising-the-face; F4, Sensitivity to others; ECQ, Empathy Components Questionnaire. Variables excluded from this model were Rutherford ToM, Rutherford control questions, Gender, and F4 from the ToMQ30. Only F1–F3 of the ToM30Q predicted empathy (DV = ECQ)*.

We can now turn to the important issue of whether the ToM30Q gives findings on dyslexia that bolster the findings from study 1 and the Rutherford task of study 2, regarding differential ToM as a function of having dyslexia. Table 6 summarises mean scores for the factors of the ToM30Q, according to gender and dyslexia status. The trends summarised in the table were analysed using a three-way mixed-model ANOVA with ToM30Q score as the dependent variable. The within-subjects factor was ToM30Q component, with four levels corresponding to our four factors from the ToM30Q. The two between-subjects factors were dyslexia status (dyslexia vs. non-dyslexia) and gender (females vs. males).

**Table 6:** Summary of ToM30Q factors by gender and Dyslexia Status.

	**F1**	**F2**	**F3**	**F4**	**Overall**
Non-Dyslexic Female	3.106 (0.079)	3.295 (0.081)	3.650 (0.080)	3.417 (0.084)	3.367 (0.051)
Non-Dyslexic Male	2.870 (0.110)	2.919 (0.113)	3.359 (0.111)	3.467 (0.118)	3.154 (0.071)
Dyslexic Female	2.858 (0.136)	3.286 (0.140)	3.550 (0.138)	3.717 (0.146)	3.353 (0.088)
Dyslexic Male	2.738 (0.167)	2.814 (0.171)	3.025 (0.169)	3.175 (0.178)	2.938 (0.108)
Non-Dyslexic	2.988 (0.068)	3.107 (0.069)	3.504 (0.068)	3.442 (0.072)	3.344 (0.045)
Dyslexic	2.798 (0.108)	3.050 (0.111)	3.288 (0.109)	3.446 (0.115)	3.251 (0.070)
Female	2.982 (0.079)	3.290 (0.081)	3.600 (0.080)	3.567 (0.084)	3.360 (0.044)
Male	2.804 (0.100)	2.867 (0.103)	3.192 (0.101)	3.321 (0.107)	3.145 (0.070)
Overall	2.893 (0.064)	3.079 (0.065)	3.396 (0.064)	3.444 (0.068)	3.203 (0.041)

*ToM, Theory of Mind. F1–F4 are factors from the ToM30Q. F1, Perceptual-based-ToM; F2, Mental-representational-ToM; F3, Prioritising-the-face; F4, Sensitivity to others. Values in parentheses are standard errors*.

Table 6 suggests scores tended to differ according to ToM30Q factor (Duval et al., 2011). The overall difference was statistically-significant [*F*_(3, 267)_ = 20.171, *p* < 0.001, Partial Eta^2^ = 0.185, Obs.Power = 1.000].

Table 6 additionally reconfirmed the tendency for females to have higher scores on the ToM30Q than did males (see also earlier r value for gender in Table 4), with this difference again significant [*F*_(1, 89)_ = 14.492, *p* < 0.001, Partial Eta^2^ = 0.140, Obs.Power = 0.964].

However, the very slender difference between dyslexic and non-dyslexic group was not statistically-significant [*F*_(1, 89)_ = 1.944, *p* = 0.167, Partial Eta^2^ = 0.021, Obs.Power = 0.281]. None of the two-way or three-way interactions were statistically-significant [Gender × ToM category—*F*_(3, 267)_ = 1.071, *p* = 0.362, Partial Eta^2^ = 0.012, Obs.Power = 0.289; Gender × Dyslexia Status—*F*_(1, 89)_ = 1.494, *p* = 0.225, Partial Eta^2^ = 0.017, Obs.Power = 0.227; Dyslexia Status × ToM Category—*F*_(1, 89)_ <1; Gender × Dyslexia Status × ToM Category—*F*_(3, 267)_ = 1.616, *p* = 0.186, Partial Eta^2^ = 0.018, Obs.Power = 0.423].

This analysis reconfirmed the gender association with the ToM factor in the above correlational analyses, as well as being in line with the oft-reported finding of higher ToM in girls beyond childhood (Ahmed and Miller, 2011; Gardner et al., 2012; Wacker et al., 2017). However, importantly and in stark contrast to our Rutherford task and our computer-based false-belief tasks (study 1), the ToM30Q (present study) did not support the contention that there exist differences in ToM according to dyslexia, neither on its own as a main effect nor in interaction with one or both of the other independent variables analysed here (Gender and ToM category).

## General Discussion

We found in study 1, that dyslexic participants performed less well on false-belief ToM questions; and that this finding was replicated with a different false-belief task (Rutherford stories task) in study 2. However, with the new ToM30Q we introduced as the main focus of study 2, we greatly reduced the reliance in particular on memory and language for working out test answers. Our findings now showed that persons having dyslexia do not in fact have any deficit at all in ToM. Below we consider these and our other findings in terms of lessons from dyslexia to ToM.

In our first study we found that the participant group previously diagnosed as having dyslexia scored lower on a false-belief task than did a non-dyslexic group. Similar findings have been reported for other neurological impairments (e.g., Tompkins et al., 2008; Sandoz et al., 2014; Ho et al., 2018; Bailey and Im-Bolter, 2020). However, this is one of the first such findings regarding ToM in adults having dyslexia.

Moran (2013) raised the possibility that ToM may function quite independently from general cognition in adults. This meant it may be possible to assess ToM whilst limiting the influence of cognitive factors known to be an issue in certain groups such as persons having dyslexia. We used this approach in study 2, in order to arrive at a new measure for ToM that was more spontaneous than in study 1. We largely avoided the need to test participants directly and relied as little as practicable on cognitive abilities such as language and memory in the ToM reasoning process. This allowed us to assess ToM as it is subjectively reflected or applied in the individual's actual real-life experiences. This pursuit was in line with the observation from many theorists, which is that ToM in real life settings may be effortless, automatic and rarely needing to become verbally explicated (Brüne and Brüne-Cohrs, 2006; Burman, 2008; Mills and Fox, 2016).

From the ToM30Q data in study 2, we extracted four factors, two of which could be termed ToM. These two ToM factors, perception-based-ToM (factor 1) and mental-representational-ToM (factor 2) exhibited lower ToM30Q scores than the remaining two factors which seemed to be more about attitudes to minds more than to the importance of reading minds (see Perner, 1991 for first distinctions of this kind).

Of these two further factors, prioritising-the-face (factor 3) seemed to be about being more interested in mental states if the mental states to be appreciated, can be partly gleaned by looking at a person's face. Note, the distinction between this factor and factor 1, is that factor 1 uses one's own emotions to assist the discerning of others mental states, unlike factor 3 which seems to require direct perception of the face itself (i.e., a perceptual cue—Wright and Dowker, 2002).

The final factor of sensitivity-towards-others (factor 4), seemed to be about being comfortable with the need to read minds during social interaction. Thus, this factor was not so much about ToM, as it was about sensitivity to the need or the potential advantages of mindreading to social interaction.

Our two ToM factors bear some resemblance to a category of ToM quite recently termed cognitive ToM; and our remaining two factors seem to resemble a second proposed category termed effective ToM (Brothers and Ring, 1992). In our study 2, individuals were found to score lower on the ToM factors (factors 1 & 2) than on the attitudinal factors (factors 3 & 4). This finding seems to imply that thinking in terms of mental states is in some sense more demanding than responding to other people's in terms of positive/negative effect (Maszk et al., 1999; Duval et al., 2011).

**Figure 1:**
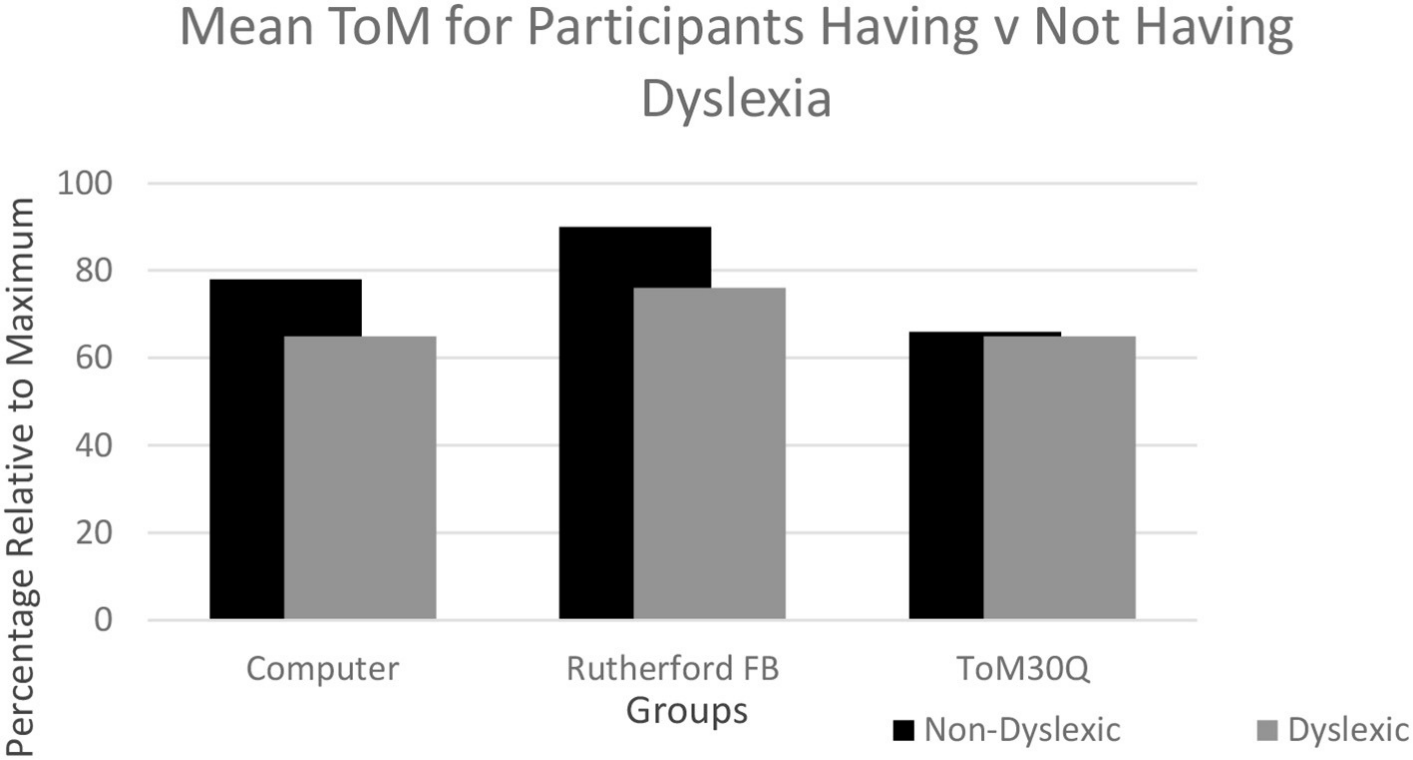
Summary According to Task and Dyslexia Status: There was a difference between ToM for participants having dyslexia vs. those not having dyslexia for the computer false-belief task and the Rutherford false-belief task. However, when the total on the ToM30Q was used, this showed no difference between the two groups. FB refers to false-belief tasks. ToM30Q includes all 4 factors.

The ToM30Q revealed the expected effects regarding correlations with empathy (Blair, 2005; Chapman et al., 2006; Singer, 2006; Lam and Yeung, 2012; Christov-Moore et al., 2014). Unlike in study 1, we observed the expected relationship to gender (Ahmed and Miller, 2011; Gardner et al., 2012; Meneghetti et al., 2012; Ibanez et al., 2013; Wacker et al., 2017). Also as anticipated, we observed a difference in profile between the ToM30Q and a second task that measured false-belief (Rutherford, 2004). The ToM30Q also showed some degree of independence between the four factors we extracted (Moran, 2013). Despite the relative success of our multiple partial validations, the ToM30Q revealed absolutely no evidence of any difference between persons having dyslexia vs. those not having dyslexia. This null result occurred both for the ToM factor and the emotionality factor. The contrast between the false-belief tasks and our ToM30Q can be seen most clearly in Figure 1. This shows no difference in ToM on the ToM30Q but a relatively robust difference on both false-belief tasks (one used with each sample).

Our findings from the ToM30Q are in line with findings from a group of African-American children assessed via a composite of three measures of ToM, which included false-belief. There were no differences between the ToM of children of low vs. high socio-economic class, there was no suggestion of differences compared to studies of the majority ethnic group (e.g., middle class White children), and no indication that basic linguistic skills (e.g., dialect differences, vocabulary differences) were associated to ToM (Longobardi et al., 2016).

The present findings are also in line with recent research concerning ADHD, which is often said to be strongly associated with dyslexia (Abdel-Hamid et al., 2019). So, we are in the position where we would have reported an apparent-dyslexia disadvantage when we use a false-belief task of ToM (computer-based false-belief task and also Rutherford stories task). However, we find not even a hint of a dyslexia disadvantage when we assess ToM in a way that greatly reduces reliance on language; despite the performance on cognitive and performance tests supporting the greater difficulties our dyslexic groups are expected to have (Moran, 2013). What this says to us is that lower performance on a false-belief ToM task does not necessarily prove lower understanding of others' minds (Bloom and German, 2000; Sandoz et al., 2014).

We draw on two additional studies in support of our view. First, McKinnon and Moscovitch (2007) found that older adults did worse on a ToM task and also on a non-ToM task, as compared to younger adults. But rather than concluding that we lose the ability to understand beliefs, intentions etc. as we mature in age, McKinnon and Moscovitch accepted the far more reasonable conclusion that computational processes that happen to be called on as part of ToM reasoning, and not necessarily ToM understanding itself, are what decline in older adults (e.g., manipulating symbolic representations in WM). Second, Marschark et al. (2000) found that when a narrative task is used instead of a false-belief task of ToM, with ToM calculated by scoring the spontaneous use of mental state attributions from the recordings, deaf children (often considered to have a ToM deficit when false-belief tasks are used—e.g., Russell et al., 1998), now do not show any ToM deficit at all, as compared to hearing children matched for age (see also Courtin, 2000; Meristo et al., 2007; Bailey and Im-Bolter, 2020).

As confirmed by taking our two studies together, it is possible to infer reasons why certain groups might appear to have a ToM deficit, when in fact they do not have any such deficit. Our explanation is in line with that of Longobardi et al. (2016) who concluded that there is a distinction between having ToM and demonstrating it via false-belief tasks. We could liken this difference to one of ToM competence vs. ToM performance. It is our view that our findings with the ToM30Q, may carry implications about the ecological validity of relying too heavily on any one particular ToM task (e.g., the standard false-belief task) when we are carrying out research that might have far-reaching implications to a particular atypical group, should the findings indicate a deficit. For example, there may be implications to strategies teachers might use to educate certain child groups, or even implications to the potential lowering of academic expectations of those groups (Simmons and Singleton, 2000; Jeffries and Everatt, 2004; Abd Ghani and Gathercole, 2013; Demetrious and Spanoudis, 2018; Bailey and Im-Bolter, 2020).

Atypicalities concerned, might include deafness, schizophrenia and potentially even autism (Gregory et al., 2002; Meristo et al., 2007; Wolf et al., 2010; Hobson, 2014; de Vaan et al., 2018; Németh et al., 2018; Acosta et al., 2019). Our thesis that ToM might be assessed without the need to actually “test” participants on false-belief tasks, does not automatically exclude the thesis that language assists the setting up of mental tokens for things in the external world and hence may be related to ToM for that reason (Astington and Jenkins, 1999; de Villiers and Pyers, 2002; Mills and Fox, 2016). Rather, it may be that language assists ToM in some respects but is an obstacle to accurately assessing ToM in certain contexts (Milligan et al., 2007; Guajardo and Cartwright, 2016). For example, language at the symbolic level may aid the setting up and maintenance of mental representations of the contents of others' minds, and hence may help make ToM an enduring ability of long-term social benefit, rather than merely a transient ability. One way of conceptualising this position is to argue that ToM may be a predictor of complex linguistic discourse skills or a mediator between basic language (e.g., production of mental state words or size of vocabulary) and spontaneous use of social narrative language (Mills and Fox, 2016; Kim, 2020). Indeed, ToM may even be predictive of written compositions (Kim, 2020). Testing all possible relationships was beyond the scope of the present paper, but we do hope to provide evidence on this in the near future.

It is also important to be aware that we are not at all advocating the abandonment of false-belief tasks. Rather, we are advocating the use of false-belief tasks alongside other tasks less reliant on memorising and linguistically (symbolically) processing multiple representations in mind. It is by using the contrast between two rather different tasks satisfying our criterion of language-diversity, and by using these tasks with the same participants (i.e., in study 2) that we have been able to establish exactly why it might seem that persons having dyslexia may have an apparent-ToM deficit, when in fact there is no such ToM deficit in dyslexia at all. Any apparent deficit in ToM in dyslexia would seem due to language-related issues rather than ToM-related issues.

Perhaps now is a good time to re-explore the possibility of ToM deficits in several atypical groups with a more diverse set of ToM tasks than used thus far. One atypical group difficult to adequately assess using variants on the standard false-belief tasks is in blindness (Roch-Levecq, 2006). It has proved highly problematic to design a physical ToM task for this group, and this may be why findings suggest that there may be a greater delay in acquiring ToM for blind children than for any other group, possibly including children having ASD (e.g., see Peterson et al., 2000). According to Hobson (2014) there are good reasons why this finding might in fact be correct. One example Hobson discusses is that low birth weight can lead both to blindness and ASD de Vaan et al. (2018). The ToM30Q, on the other hand, would be as relevant to assessing ToM in blind participants as it is to sighted participants; and hence it promises to more definitively answer this question.

Indeed, the ToM30Q even raises the possibility of assessing young children who fail the standard false-belief task, for example by asking their main caregiver to answer the questions on the ToM30Q on behalf of their child. This carries the further benefit that it can be used even in the current Covid-19 climate, because testing can be done on a pseudo face-to-face basis via platforms such as Zoom or MS-Teams, by using only the audio channel on such a platform, or even less directly by using platforms such as Qualtrics.

Finally, we acknowledge potential issues with our studies that should be borne in mind alongside our very positive findings. One is that we relied on slightly < 100 participants in each of our two studies. However, many other studies have produced meaningful findings with similar or smaller sample sizes than here (Astington and Jenkins, 1999; Meristo et al., 2007; Mills and Fox, 2016; Bailey and Im-Bolter, 2020). Another potential issue is that our factor analysis of the ToM30Q in study 2 might be considered less robust for reasons of sample size. Although this will of course be true, we believe it might actually render our three partial validations all the more persuasive. A third potential issue is that the reliability estimate for study 2 was quite low compared to in study 1. This may have been due to us needing to rely on only one of the four stories from the Rutherford task we used as part of study 2.

## Conclusions

Dyslexia initially was found to be associated with lower ToM performance as indexed by a computer-based and a non-computer-based false-belief task. However, we then in some sense controlled for language, performance issues and other cognitive issues that could affect performance on experimental tasks but which might not be integral to ToM itself. Our resultant task, based around extraction of at least two ToM factors from a 30 item questionnaire about what reasoners feel is important in their interactions with others, showed that any difference in ToM performance between a dyslexic and non-dyslexic group completely vanished.

All of the four factors on the ToM30Q (two relating to cognitive ToM and two more attuned to attitude to presence of other minds) were quite valid and reliable. For example, we replicated previously reported profiles by gender and associations with the ECQ questionnaire on empathy. We also found partial validation in terms of the factor we called prioritising-the-face (the first of the two attitudinal/effective factors—factor 3), when the comparator for our ToM30Q was the Rutherford stories task.

Despite our very encouraging findings regarding dyslexia in this research, we would of course concede that ours is an initial exploratory study. It is therefore necessary to further confirm the utility of the ToM30Q with wider studies with other participant groups and also a variety of socio-cognitive phenomena. We are in the process of providing such studies. However, our present conclusion regarding dyslexia, that experimental tasks of ToM such as the false-belief task may tend to confound cognitive domains such as language with the ability to think in terms of minds itself, does seem plausible on our present findings. Dyslexia does not involve a deficit in ToM, but over-reliance on memory and verbal reporting and false-belief tasks may make it seem so.

## Data Availability Statement

The raw data supporting the conclusions of this article will be made available by the authors, without undue reservation.

## Ethics Statement

The studies involving human participants were reviewed and approved by Department of Life Sciences Research Ethics Committee, Brunel University. The patients/participants provided their written informed consent to participate in this study.

## Author Contributions

The authors each contributed to planning and data analysis of the research, and writing up of the manuscript.

## Conflict of Interest

The authors declare that the research was conducted in the absence of any commercial or financial relationships that could be construed as a potential conflict of interest.
