# Supplementary material for: Language Can Obscure as Well as Facilitate Apparent-Theory of Mind Performance: Part 2—The Case of Dyslexia in Adulthood
Source: Front Psychol. 2021 Jun 24;12:621457. doi: 10.3389/fpsyg.2021.621457 (PMC8264364; doi:10.3389/fpsyg.2021.621457)
Supplement: Supplementary file 1 [file Data_Sheet_1.pdf]

Appendix 1

Scenario 4 – Going Out

Three friends Mark, Amy and John are out at a night club in town.

They are having a great time but decide to take a break from dancing as they are all hot and tired.

Mark decides to go to the bar and get some drinks for himself and his 2 friends.

The other 2 are sat down waiting for Mark to come back with the drinks.

At the bar Mark orders 3 alcoholic drinks, one with coke and two with blackcurrant.

The drink with coke is for himself, because he dislikes blackcurrant.

After putting the three drinks down he goes off to the toilet.

John decides to play a joke on Mark by switching drinks with his own.

However, Mark had inadvertently given John his drink with coke, instead of one of the drinks with blackcurrant.

Amy and John are laughing about what Mark will do when he finds out.

They will wait until he gets back so they can all try their drinks at the same time.

Questions on Scenario 4

1, How does John think Mark will feel upon tasting his drink? *2nd Order*

2, What drink did Mark place in front of John? *Factual*

3, Which drink will John assume he will taste? *1st Order*

4, How many drinks did Mark buy? *Factual*

5, What drink does Mark think he will have when he gets back from the toilet? *1st Order*

6, Which drink does John think Mark brought back for him? *Inference*

7, What drink did Mark buy for Amy? *Inference*

8, What drink does John think Mark will taste? *2nd Order*

Appendix 2

Table 1b: Summary of Tests of Spelling, WM and Reading Raw Scores (Study 1)

	Spelling	WM	Reading	Overall
Non-Dyslexic Female	43.329 (0.693)	11.371 (0.218)	51.000 (0.466)	35.234 (0.338)
Non-Dyslexic Male	41.318 (0.895)	11.190 (0.281)	51.524 (0.602)	34.677 (0.436)
Dyslexic Female	35.814 (0.874)	10.182 (0.275)	47.455 (0.588)	31.150 (0.426)
Dyslexic Male	36.182 (1.236)	10.364 (0.389)	45.727 (0.832)	30.758 (0.603)
Non-Dyslexic	42.324 (0.566)	11.281 (0.178)	51.262 (0.381)	34.955 (0.276)
Dyslexic	35.998 (0.757)	10.273 (0.238)	46.591 (0.509)	30.954 (0.369)
Female	39.572 (0.558)	10.777 (0.175)	49.227 (0.375)	33.192 (0.272)
Male	38.750 (0.763)	10.777 (0.240)	48.626 (0.513)	32.717 (0.372)
Overall	39.161 (0.473)	10.777 (0.149)	48.926 (0.318)	32.955 (0.231)

Note: Values represent raw scores. Values in Parentheses are standard errors.

Appendix 3

Appendix 3: Summary of Items for Factors on the ToM30Q			
**Factor 1**		**Factor 2**	
Questions	Factor Level	Questions	Factor Level
18. Are you ever really an emotional person?	.688	10. After you have just won an argument with someone, do you try to think about how the other person might be feeling?	.733
12. When someone is facing challenging or difficult times, are you kind of person to get a bit emotional.	.620	16. When someone does something do you try to imagine what they were thinking that made them do it.	.612
25. To what extent do people need to know how someone was feeling in order to understand how they acted?	.609	3. After a disagreement with someone else has been resolved in my favour, I stop thinking about how that person might be feeling.	.611
24. Often, we can tell what someone is thinking just by looking into their eyes.	.605	7. Do you find it hard to relate to people who are suffering from things that you have never experienced yourself?	.608
26. Can you easily tell what someone is going through from the way they are behaving?	.604	8. Is it important to appreciate how people were felling, when one is trying to make sense of how they behaved?	.599
22. People's eyes usually tell the truth.	.576	28. When thinking about what someone is going through, it is important to try and put myself in their shoes.	.557
9. I believe that my past views about something are often the same as views I currently hold about it.	.547	11. We should judge what people do rather than what make them do it.	.471
17. When talking someone over the phone, it is hard to quickly tell when they are just beginning to get upset.	.481		
**Factor 3**		**Factor 4**	
Questions	Factor Level	Questions	Factor Level
30. In a face to face conversation with friends, I am one of the first to be able to tell that someone is getting upset.	.682	21. The tone of someone's voice says very little about how they are feeling.	.662
13. In a face to face conversation with friends, I am one of the last to be able to tell that someone's mood is changing.	.674	23. When in conversation with someone, it is fine if I cannot tell how other people are feeling.	.645
19. I am poor at giving face to face advice to people who are in need.	.664	1, In your view how often do people do things for no reason	.463
5. Is it generally easy to people to notice when they have done something to upset someone else	.409	2, Knowing what other people are thinking is important to me	.455

Appendix 4

Appendix 4: Summary of Items for Single Factor on the ECQ			
Factor 1		Excluded	
Questions		Questions	
21. When someone is crying, I tend to become very upset myself	-.760	24. I am not very good at noticing if someone is hiding their emotions	-
7. I am not very good at helping others deal with their feelings	.749	1. I am usually successful in judging if someone says on thing but means another	-
2. When someone seems upset, I am usually uninterested and unaffected by their emotions	.703	3. I am not very good at predicting what other people will do	-
5. I am good at responding to other people's feelings	-.665	8. Others' emotions do not motivate my mood	-
9. I have a desire to help other people	-.628	27. I take an interest in looking at both sides to every argument	-
16. I am uninterested in putting myself in another's shoes if I am upset with them	.601	15. I like to know what happens to others	-
19. I like trying to understand what might be going through my friends' minds	-.588	25. During a conversation, I'm not very good at figuring out what others might want to talk about	-
13. I avoid getting emotionally involved with a friend's problems	.579	26. I am good at sensing whether or not I am interrupting a conversation	-
6. I am not interested in protecting others, even if I know they are being lied to	.578		
23. I avoid thinking how my friends will respond before I do something	.562		
4. My friends often tell me intimate things about themselves as I am very helpful	-.556		
11. I feel pity for people I see being bullied	-.555		
22. I don't intuitively tune into how others feel	.548		
12. I strive to see how it would feel to be in someone else's situation before criticizing them	-.546		
10. When talking with others, I am not very interested in what they might be thinking	.526		
18. I am not always interested in sharing others' happiness	.522		
17. When I do things, I like to take others' feelings into account	-.511		
20. I am poor at sharing emotions with others	.469		
14. I do well at noticing when one of my friends is uncomfortable	-.451		
